# The Impact of Arthritis on Canadian Women

**DOI:** 10.1186/1472-6874-4-S1-S18

**Published:** 2004-08-25

**Authors:** Elizabeth M Badley, Naomi M Kasman

**Affiliations:** 1 Department of Public Health Sciences, University of Toronto, Toronto, Canada; 2 University Health Network, Toronto, Canada

## Abstract

**Health Issue:**

Arthritis is one of the most prevalent chronic conditions in Canada and a leading cause of long-term disability, pain, and increased health care utilization. It is also a far more prevalent condition among women than men. Information was obtained primarily from the 1998–99 National Population Health Survey and the Canadian Joint Replacement Registry.

**Key Findings:**

In 1998, the overall prevalence of self-reported arthritis or rheumatism in Canadian women was 20.0%. This rate increased to 55.6% among women over 75 years of age. Compared to women with chronic conditions, women with arthritis were more likely to experience long-term disability; report worse health; experience more pain; be dependent upon others and consult general practitioners, specialists, and physiotherapists more frequently. While men and women with arthritis under-utilize total joint replacement surgery, the degree of under-use was over three times greater for women.

**Data Gaps and Recommendations:**

There is a lack of detailed information on the use of health care services by women with arthritis. There are also no systematic data available on the prescribing of medications, access to services such as assistive devices or exercise programs, or use of community support, self-management strategies, or rehabilitation services. The burden of arthritis both on women and on society is expected to increase as the population ages. A comprehensive health strategy to reduce the impact of arthritis is required to ensure that health and support services are available in a timely manner and provided in such a way to meet the needs of Canadian women.

## Background

Arthritis is currently one of the most prevalent chronic conditions in Canada and is a leading cause of long-term disability, pain, and increased health care utilization[1,2] The term "arthritis" is defined as "inflammation of the joints," and it encompasses over 100 different conditions. Some conditions included under the "arthritis and rheumatism" designation are osteoarthritis, rheumatoid arthritis, fibromyalgia and systemic lupus erythematosus. The most common symptoms of arthritis are joint pain, stiffness, swelling and muscle weakness; these symptoms can lead to joint damage, which may limit the range of movement and/or deform the joint [3-5].

Arthritis is a far more prevalent condition among women than among men [6-10]. Findings from numerous studies have shown that rheumatoid arthritis affects up to 2.5 times as many women as men and that the female:male ratio for osteoarthritis is approximately 2:1[9,11-16]. While there is currently no known cure for arthritis or rheumatism, there are various treatments available, including drugs, surgery, rehabilitation and self-management, that have been shown to prevent long-term disability, maintain function and reduce the pain that is associated with these conditions[11].

This chapter presents information on the status and impact of arthritis and rheumatism on women in Canada. The impact of the disease will be measured in terms of ill health, pain, long-term disability and health care utilization. In order to set the data on arthritis in women in a broader population health context, we also present data on men with arthritis and various subgroups of women, including those with chronic conditions other than arthritis and those who report no chronic conditions. The information presented in this report has been obtained primarily from analysis of data from the 1998–1999 National Population Health Survey (NPHS) as well as from the Canadian Joint Replacement Registry.

## Methods

The analyses in this chapter are based on the cross-sectional household data from the third cycle (1998–1999) of the NPHS (see Appendix A). Data from the health file for the 14,682 selected respondents who were over the age of 15 were weighted – taking into account the sample design, adjustments for non-response and post-stratification – to represent about 23.8 million Canadians.

Arthritis was identified in the NPHS by asking respondents whether they had arthritis or rheumatism as a long-term chronic condition (defined as a condition diagnosed by a health professional that had lasted, or was expected to last, six months or more). Individuals were also asked early in the survey to report their sex (male/female); answers to all subsequent questions could thus be linked to each individual's response.

The Canadian Joint Replacement Registry (CJRR) is a fairly new, national registry (launched in June 2000) that collects information on total hip and total knee replacement surgeries performed in Canada and follows joint replacement recipients over time to monitor outcomes. The CJRR is a joint effort between the Canadian Institute for Health Information (CIHI) and the orthopaedic surgeons of Canada.

## Results

### Demographic Characteristics

According to data from the 1998–1999 NPHS, almost 4 million individuals over the age of 15 reported having arthritis that had been diagnosed by a health professional, two thirds of these individuals (approximately 2.4 million) being women. This represents a disease prevalence among women of 20%, which was almost double the percentage of men who reported the condition (12%).

**Figure 1:**
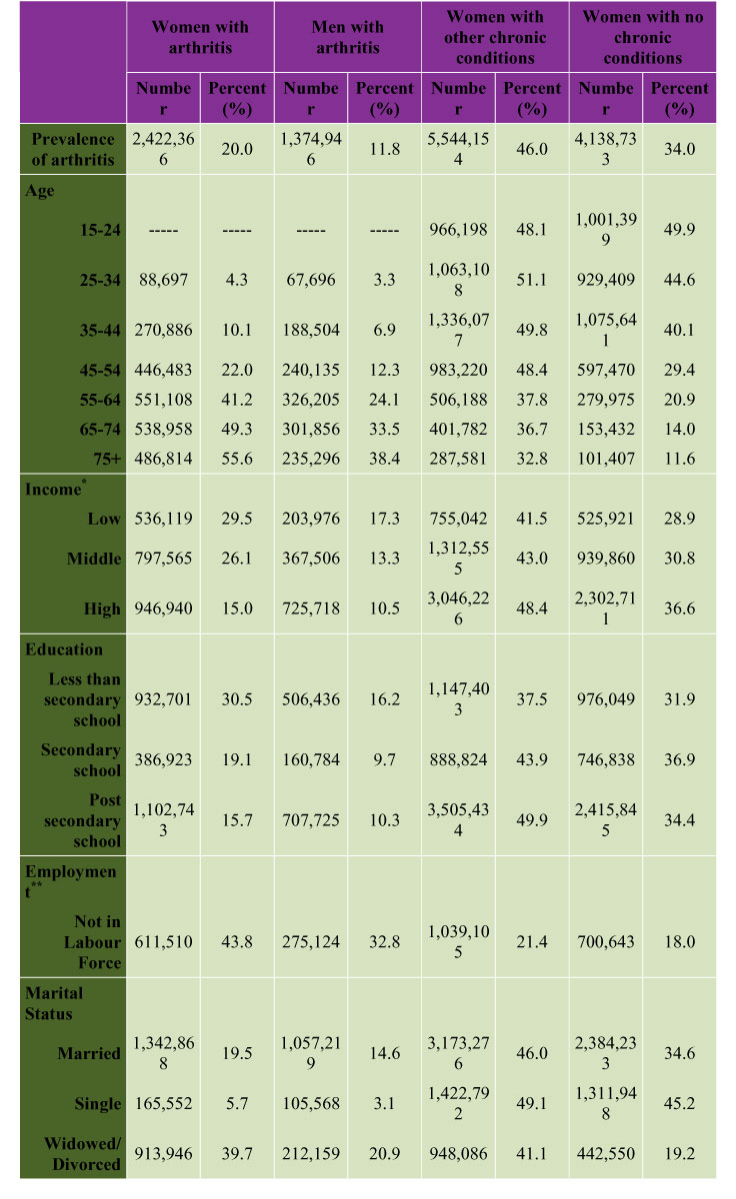
Socio-Demographic Characteristics of 1998/99 NPHS Respondents, Stratified by Sex and Disease Status

**Figure 2:**
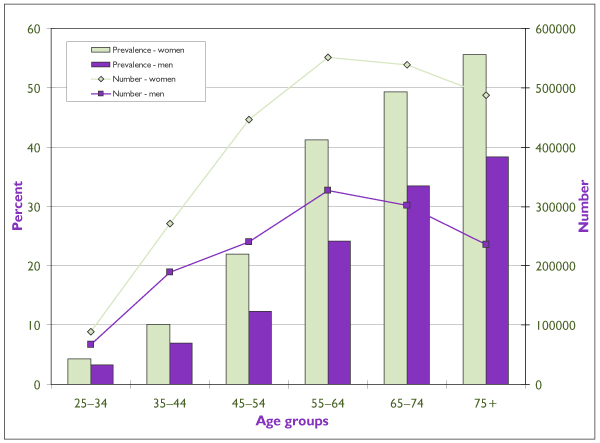
**Number and Prevalence of Self-Reported Arthritis or Rheumatism, by Age and Sex **Source: NPHS, Statistics Canada, 1998–1999

The rates of arthritis increased with age among both men and women (Figures 1 and 2), resulting in a maximum disease prevalence in the 75 and older age group for both sexes. Women displayed a higher prevalence rate of arthritis than men within each age group. More than 55% of the women over the age of 75 reported that they had arthritis, which represents almost half a million women in the Canadian population and is more than double the number of men in the same age group who were affected. Despite the consistent increase in prevalence rates with age, the actual number of individuals with arthritis peaks in the 55 to 64 age group and then begins to decline in the older age groups, a result of the age structure of the Canadian population. In fact, 60% of individuals who reported arthritis or rheumatism were between the ages of 15 and 64. In contrast to that of arthritis, the age distribution among women who reported other chronic conditions reached their peaks at a younger age (25 to 34) and was followed by a subsequent decline in prevalence rates. As might be expected, the proportion of women who reported having no chronic conditions also declined dramatically with increasing age.

**Figure 3:**
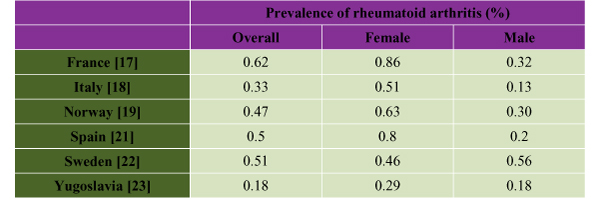
International Prevalence Rates of Rheumatoid Arthritis by Sex

Few studies have reported international comparisons of arthritis prevalence, and those that have been carried out have mainly reported on rheumatoid arthritis (for which there are internationally accepted criteria for diagnosis) and not osteoarthritis. Figure 3 displays some of the published sex-specific international prevalence rates for rheumatoid arthritis [17-22]. Ethnicity has also been looked at with respect to arthritis prevalence, and higher rates of arthritis have been seen in many ethnic groups, including Native Canadians, Native Americans and African Americans[13,15].

### Socio-Economic Status

**Figure 4:**
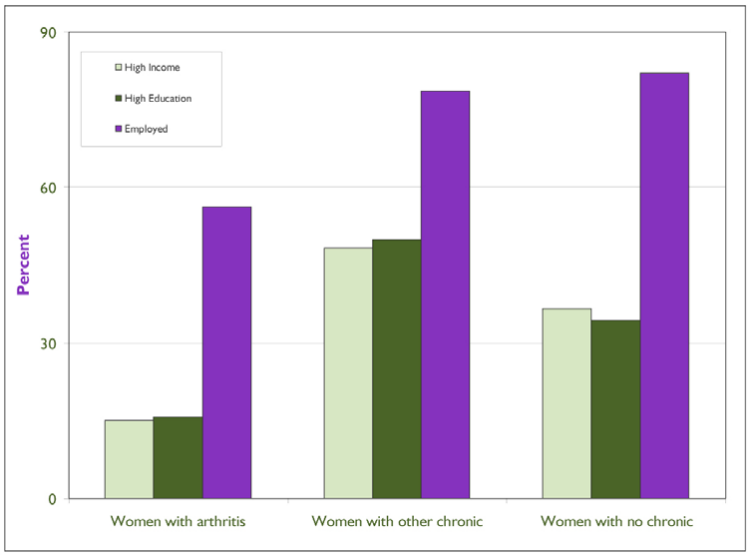
**Percentage of Women in Income, Education, And Employment Classes, by Disease Status **Source: NPHS, Statistics Canada, 1998–1999

Socio-economic status is a concept that is generally measured by income and by education and/or employment variables. Arthritis is a disease that affects individuals from all socio-economic classes. However, a higher percentage of women who reported low or middle income and fewer years of education reported arthritis than did women from higher socio-economic classes (Figure 1). For example, 30% of women in the lowest income category reported arthritis, which was double the percentage of women with high income who reported these conditions. Similarly, income and educational disparities were seen in men with arthritis, although these differences were far less marked. The opposite relation held for chronic conditions other than arthritis: women who reported high income or a post-secondary education were the most likely to also report a chronic condition (Figure 4). Women with high income were also more likely to report no chronic conditions.

In terms of marital status, 40% of women who were widowed or divorced reported the presence of arthritis, whereas less than 6% of single women did so. This is likely a function of the age distribution of women with arthritis. A substantial proportion (44%) of working-age women (between 16 and 64 years of age) with arthritis and 33% of men with arthritis reported not currently being in the labour force, as compared with the 21% of women who reported chronic conditions other than arthritis and 18% of women who reported no chronic conditions.

### Long-Term Disability and Health Status

**Figure 5:**
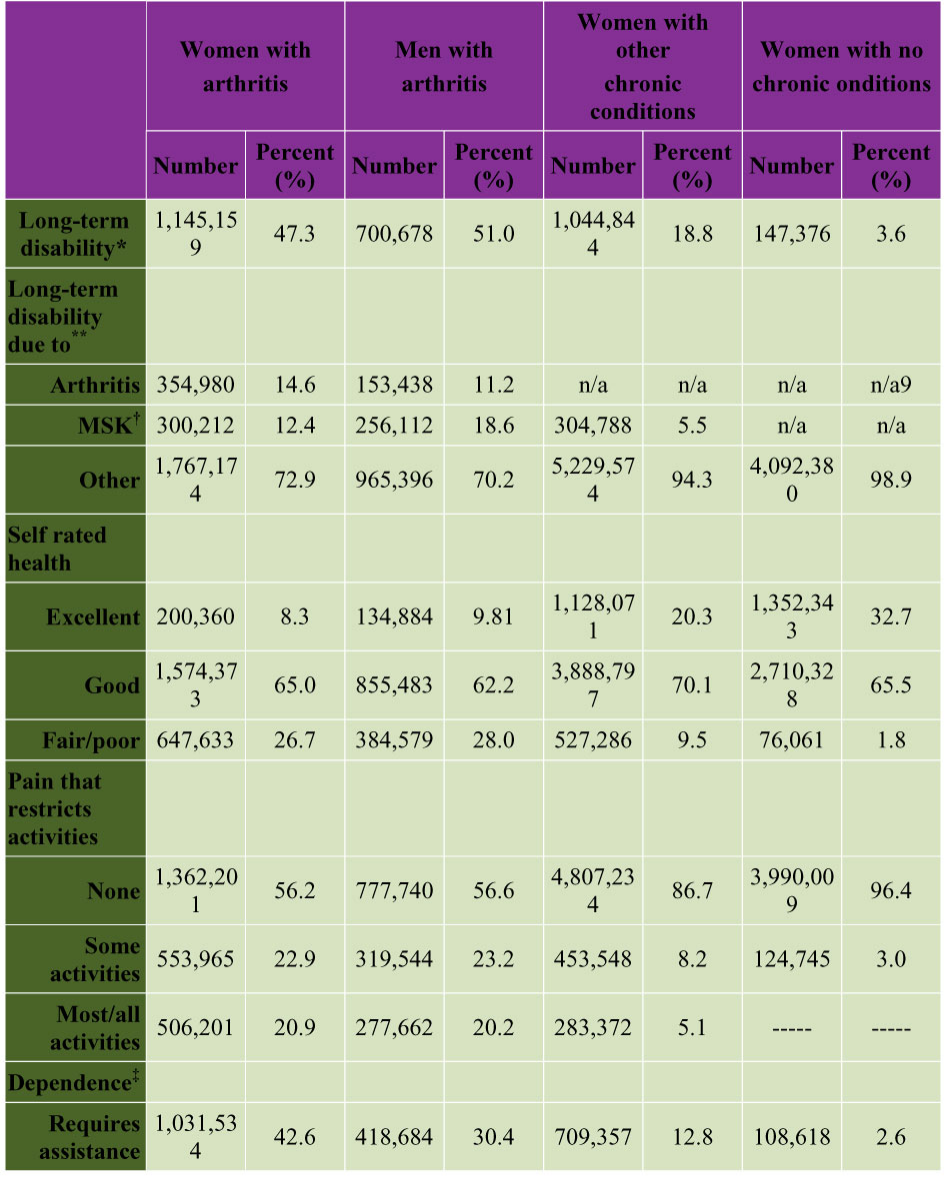
Health Status Measures of 1998/99 NPHS Respondents, Stratified by Sex and Disease Status

**Figure 6:**
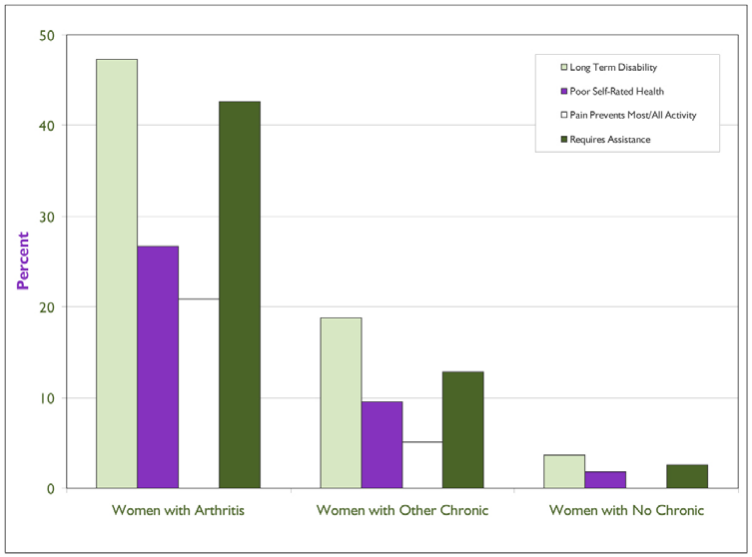
**Percentage of Women With Health Impacts, by Disease **Status Source: NPHS, Statistics Canada, 1998–1999

Arthritis has a major impact in terms of long-term disability, which is measured in the 1998–1999 NPHS as long-term activity restriction at home, at work, at school or in leisure-time activities. Approximately 50% of men and women with arthritis reported long-term disability, as compared with about 20% of women with other chronic conditions and less than 5% of women with no chronic conditions (Figures 5 and 6). While men with arthritis had a slightly higher overall prevalence of long-term disability than women with arthritis, a higher percentage of the women reported that their disability was specifically attributable to their arthritis, whereas men were more likely to attribute their disability to other musculoskeletal conditions (e.g. chronic back pain). Arthritis was also strongly associated with a report of poor self-rated health, in that more than 30% of women with arthritis reported only fair or poor health. A much lower proportion (10%) of women with chronic conditions other than arthritis and less than 2% of women with no chronic conditions rated their health as fair or poor.

Approximately 45% of women with arthritis reported pain that prevented at least some activities, and 21% reported that pain prevented them from participating in all or most activities. Rates among women and men with arthritis were quite similar in terms of the experience of pain. Women who reported chronic conditions other than arthritis or who reported no chronic conditions were far less likely to experience pain – 87% and 96% respectively reporting that no activities were limited as a result of pain.

When individuals were asked whether, because of any condition or health problems, they required assistance to accomplish a variety of tasks (including personal care, household chores, shopping, etc.), 43% of women with arthritis reported that they were dependent upon others to assist them with one or more of these activities. Only 30% of men with arthritis, less than 13% of women with other chronic conditions and 3% of women with no chronic conditions reported a need for assistance.

### Health Care Utilization

**Figure 7:**
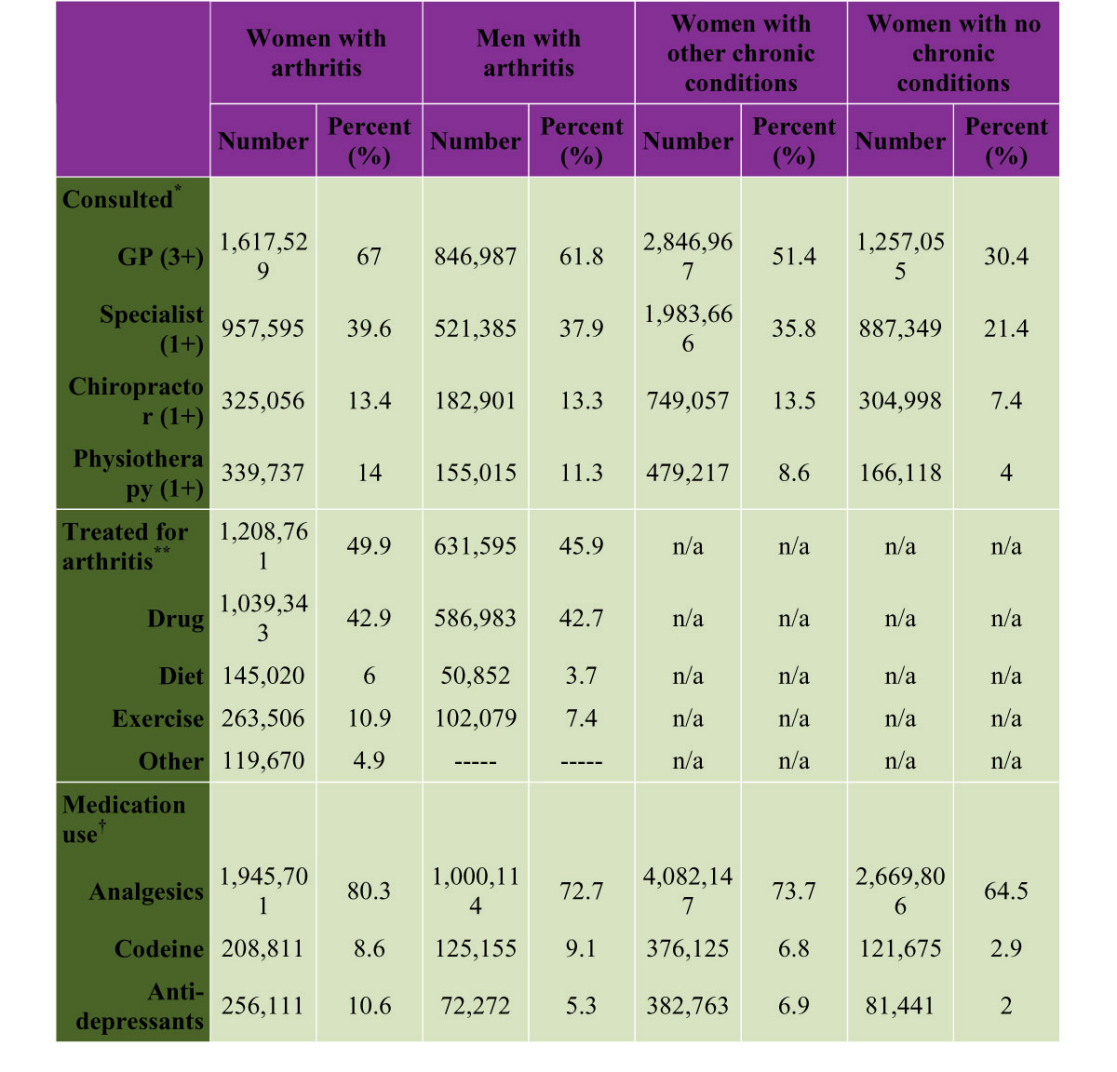
Health Care Utilization by 1998/99 NPHS Respondents, Stratified by Sex and Disease Status

**Figure 8:**
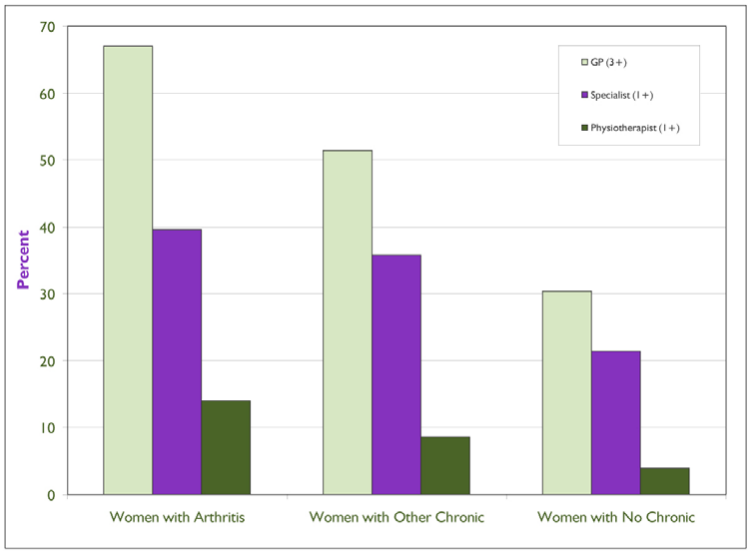
**Percentage of Women Using Health Care Services, by Disease **Status Source: NPHS, Statistics Canada, 1998–1999

A slightly higher percentage of women with arthritis (67%) than men with arthritis (62%) reported that they had consulted a general practitioner (GP) three or more times in the previous 12 months (Figures 7 and 8). For women with other chronic conditions or no chronic conditions the percentage was substantially lower, at 51% and 30% respectively. Approximately equal proportions of women (40%) and men (38%) with arthritis and women with a chronic condition other than arthritis (36%) reported that they had consulted a medical doctor other than a GP at least once in the previous year; the proportion of women with no chronic conditions who had consulted a specialist in the previous year was notably lower, at only 21%.

In terms of allied health care services, both women and men with arthritis and women with other chronic conditions were equally likely – approximately 13% in all groups – to have consulted a chiropractor at least once in the previous year. However, women with arthritis reported having visited a physiotherapist more frequently than either men with arthritis, women with chronic conditions other than arthritis or women with no chronic conditions.

When individuals with arthritis were asked specifically about what treatment, if any, they used to care for their arthritis, almost 50% of women reported that they had used some treatment, slightly higher than the 46% of males who had done so. Arthritis treatment options were further broken down into specific types. Approximately equal proportions of both sexes had used medication (43%), whereas women reported using diet and exercise more frequently than men. The 1998–1999 NPHS also asked about the use of some specific types of medications. Women with arthritis were more likely than men with arthritis and women with other chronic conditions to report the use of pain medications (80% versus 73% and 74% respectively) and antidepressants (11% versus 5% and 7% respectively) but equally likely as men with arthritis to report the use of medications containing codeine (9% for both).

**Figure 9:**
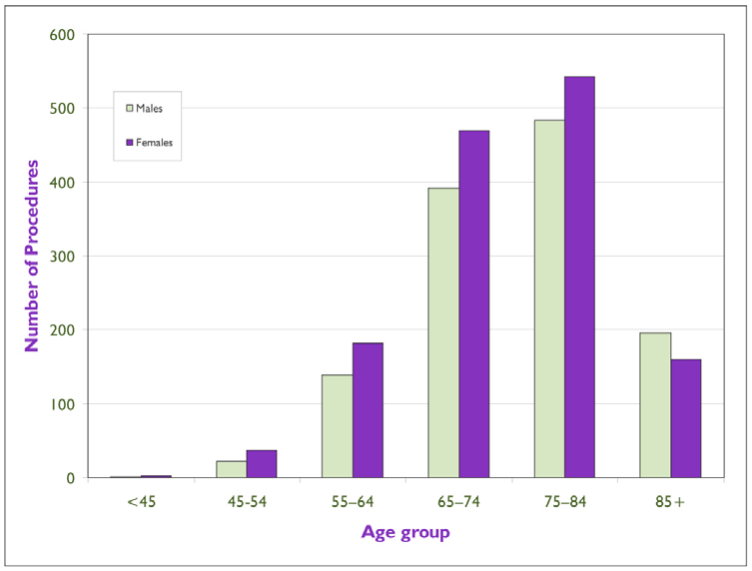
**Age Specific Rates (per 100,000 population) of Total Knee Replacement Procedures, by Sex, Canada, 1999–2000 **Source: CJRR, CIHI, 1999, 2000

Total joint replacement (TJR) surgery is a cost-effective means of treating primarily osteoarthritis. TJR can provide significant pain relief and improvement in an arthritis sufferer's functional status and quality of life[23]. According to the most recent data published in the 2002 Report of the Canadian Joint Replacement Registry (i.e. for 1999–2000), 62.2 and 73.3 women per 100,000 population had had total hip and total knee replacement surgeries respectively[24]. The age-specific rates for total knee replacements performed on both men and women in Canada for 1999–2000 can be found in Figure 9. Although these rates were generally slightly higher among females than males, this finding needs to be viewed in light of the overall prevalence rate for arthritis among women, which was almost double that among men (Figure 2).

## Discussion

### Characteristics of Women with Arthritis

Women in Canada report arthritis more frequently than men. According to the NPHS, compared with women with other chronic conditions, women with arthritis were older, had lower incomes and fewer years of education and were more likely to be widowed and out of the labour force. Consequently, it appears that the women who are experiencing arthritis may be the women with the fewest resources to deal with the impact of the condition on their daily lives. In terms of the health impacts of arthritis, women with the disease were far more likely to experience long-term disability than women with other chronic conditions. They also reported poorer health, experienced more pain that restricted activity, and consulted with GPs, specialists and physiotherapists more frequently.

### Treatment Options

The prevalence of arthritis increases so dramatically with age that many older people consider the disease to be a normal aspect of aging until it becomes sufficiently painful and/or debilitating to prompt medical care[6]. However, it is a misconception that arthritis is a disease only of the elderly, since the largest number of individuals with arthritis is in the age range 45 to 64. Another common misconception is that arthritis is an inevitable part of growing old for which nothing can be done. Although there is, at present, no known cure for arthritis, appropriate treatment helps to prevent long-term disability, maintain function and reduce pain. For some types of arthritis, particularly rheumatoid arthritis, a rheumatologist may best manage treatment[15], while total joint replacement surgery is an effective therapy for those with advanced osteoarthritis of the hip or knee[25]. As well, self-management strategies, including exercise and education, have been shown to help reduce pain and long-term disability and to decrease the need for medications[26].

### Health Care Utilization

Despite the well-documented benefits of the various available treatments for arthritis, surveys have repeatedly shown that between 40% and 60% of individuals with self-reported joint symptoms or signs of arthritis or rheumatism are not receiving treatment. The reason behind the relatively low percentage of individuals with arthritis or rheumatism who see a health professional is unknown, but rates of health care utilization have been shown to be affected by a wide variety of factors, including sex, age and socio-economic status[27].

It is difficult to make comparisons between men and women about the use of health care services for arthritis because basic sex differences in prevalence rates and/or disease severity must be taken into account. For example, despite the slightly higher rate of TJR among women, a recent Canadian study found that although there is under-use of TJR in both sexes, once disease prevalence and need are accounted for, the degree of under-use is more than three times as great in women as in men[27].

The issue of health care utilization by those with arthritis is of particular importance since recent research done in the United States has suggested that individuals with arthritis who receive appropriate care (such as care from a rheumatologist) experience lower rates of long-term disability than those who receive care on a more irregular basis[28]. If the health care services available for those with arthritis or rheumatism are not being used, particularly if a specific group of people (such as women) are not being reached, the effects of this disease may be far more debilitating than is necessary and greatly decrease quality of life. There are also issues surrounding the availability of specialized health care services, such as TJR surgery and the services of rheumatologists, in some of the more rural regions of Canada. Research is currently being conducted on regional variation in service availability, although the findings were not available for inclusion in this report.

The burden of arthritis in women also has considerable societal costs. A high proportion of women with arthritis reported not being in the labour force. This finding has been reported in other studies as well, many of which have also noted that simple and non-costly adaptations, such as flexible work schedules, may enable many people to remain in, or return to, the workplace [29-31]. As well, women often have roles as homemakers and caregivers that may be affected by arthritis disability. Since most disability and employment studies focus only on paid work, relatively little is known about the effect of arthritis on domestic responsibilities[23].

### Gaps and Recommendations

A number of gaps exist in the arthritis and gender data that are currently available. A major gap is the lack of detailed data on the use of health care services by women with arthritis. Most of the care for arthritis, including specialist care, is delivered in ambulatory settings on which there are currently no systematic data available, although, in principle, such data could be captured by a provincial database of physician billing claims. Data are available on surgery and hospitalization, but only a minority of women with arthritis undergo these interventions. There are no systematic data available on the prescribing of medications, the use of rehabilitation services, such as physical and occupational therapy, or access to other services, such as assistive devices, therapeutic exercise programs, community support and self-management. The need for data on the use of prescription medication is of increasing importance in light of current advances in the development of stomach-sparing anti-inflammatory drugs (e.g. COX-2 inhibitors) and effective, but expensive, drugs for the treatment of rheumatoid arthritis. People with arthritis are also major users of alternative health care services and herbal medications. This is a further area in which there is a deficit of reliable information about efficacy and use.

Our only sources of comprehensive population-based data are national and provincial health surveys. These surveys rely on self-reported information regarding socio-demographic characteristics, medical conditions and health status, although there are questions surrounding the validity of this method. Another issue with these surveys is that they do not enable us to differentiate between the many types of arthritis, such as osteoarthritis and rheumatoid arthritis. This is important, since patients with rheumatoid arthritis tend to report more frequent and intense pain than patients with osteoarthritis, and the proportion of individuals with rheumatoid arthritis who are women is higher than the proportion with osteoarthritis[32]. Also, data from these surveys do not allow health status or health utilization to be attributed to a specific disease. Therefore, even if an individual reports the presence of both arthritis and long-term disability, it cannot be ascertained whether the long-term disability is a direct result of the arthritis.

Information about the impact of arthritis on women and their associated use of health care services is vital to underpin a strategy aimed at reducing the impact of this disease. Such a strategy would require that health and support services be available in a timely manner and provided in such a way as to meet the needs of women. Any such strategy would need to address access to primary care physicians and specialist services. There are perceived deficiencies in the primary care management of arthritis, and areas of concern include timely referral to specialists, advice to patients about exercise and other non-pharmacological interventions, and the examination of joints [33-36]. We lack information about the use of medical specialists, particularly rheumatologists, by women with arthritis. More information is clearly needed about the factors associated with use of care by women for their arthritis.

The disability associated with arthritis often leads to reduced independence, to lower participation in employment, social and leisure activities, and to loss of income and the incurring of extra expenses. Rehabilitation therapy and community support services need to be in place to help individuals deal with the disabilities and associated costs arising from arthritis. These services represent a vast but largely uncharted territory. There are few data about them and, in the case of community services, very little documentation about what services are available and how they are used. There is evidence that the use of education and self-management strategies can lead to significant decreases in pain, disability and medical consultation as well as increases in self-efficacy [37-39]. As well, this type of "self-managed" intervention appears to be more frequently used by women than men[40]. This points to the need for further research and more investment in community-based interventions that may facilitate the day-to-day management of arthritis by women.

## Conclusion

The burden of arthritis both on women and on society is expected to increase with the aging of the population. Because of the current age structure of the Canadian population and the aging of the "baby boomer" generation (those born between 1946 and 1960), a large increase is expected in the number of people over the age of 65 and a subsequent increase of approximately 1 million per decade over the next 30 years in the number of people with arthritis or rheumatism. Sixty per cent of this increase in prevalence will occur among women. These statistics alone point to the need for a comprehensive health strategy to reduce the impact of arthritis. Major efforts are also needed to find ways of capturing information on the use of ambulatory care, specialists, medications, rehabilitation and other services by both women and men with arthritis.

Arthritis is one of the most frequent chronic conditions in women in Canada, and the health care and support systems aimed at dealing with the impact of arthritis need to be geared to the provision of care over a longer period of time, the ultimate goal of care being to improve the situation of women with arthritis and their families. It is hoped that this report will contribute to putting arthritis on the agenda of women's health.
